# Inflammatory Myofibroblastic Tumor of the Trachea with Concomitant Granulomatous Lymph Node Lesions

**DOI:** 10.1155/2011/151729

**Published:** 2011-09-13

**Authors:** Julia Anne Koch, Patrick Dorn, Thierry Rausch, Hans-Beat Ris, Hans-Anton Lehr, Stephan C. Schäfer

**Affiliations:** ^1^Department of Thoracic and Vascular Surgery, CHUV, 1011 Lausanne, Switzerland; ^2^Department of Surgery, Kantonsspital Graubünden, 7000 Chur, Switzerland; ^3^Institute of Pathology, CHUV, 1011 Lausanne, Switzerland

## Abstract

We report herein the case of a 57-year-old lady who had two concomittant lesions, an inflammatory myofibroblastic tumor in the trachea, and severe granulomatous lesions in the adjacent hilar lymph nodes. While these two lesions shared histological and some immunohistochemical features lesions. They differed in terms of ALK-1 expression, which was positive in the tracheal tumor and negative in the lymph nodes. The discussion of the case circles around putative pathophysiological links between the lesions. The authors favor the idea that the lymph nodes present a sarcoid-like granulomatous reaction to the inflammatory myofibroblastic tumor in the trachea over a coexistence of two independent entities. However, no conclusive evidence for this interpretation can be presented based on the existing literature.

## 1. Case Report

**Figure 1:**
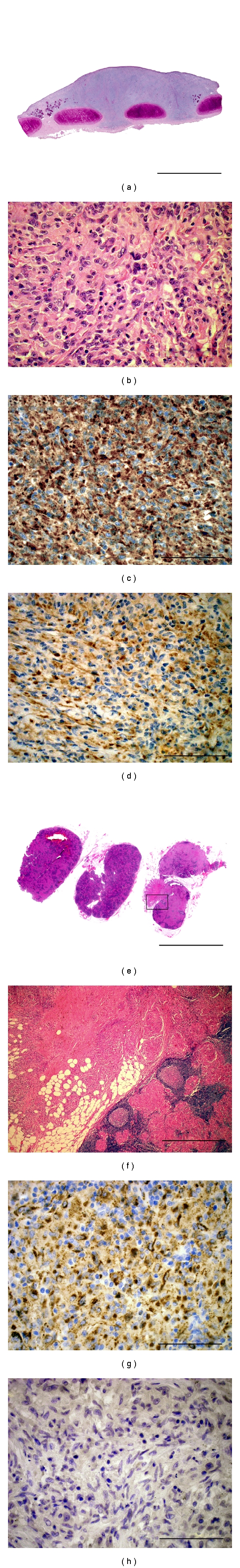
Representative image of the inflammatory myofibroblastic tumor (IMT) in the distant tracheal portion in a 57-year-old lady. (a) Overview of the lesion spanning three cartilage rings (PAS). (b) Note the partly spindly architecture of the lesion, with only very mild nuclear atypia and absent mitotic activity (H&E). Immunohistochemical documentation of CD68 expression (clone PGM1, (c)) and of ALK1 expression (d). The lower four panels (e)–(h) depict representative images of one of 15 peritracheal lymph nodes in the vicinity of the tracheal lesion shown in panel (a). (e) Overview over the deep sections through the lymph node, which shows that the granulomatous lesions largely replaces the lymph node and focally transgresses its capsule (H&E). (f) Higher power magnification of the marked area in (a). Note the striking granulomatous reaction replacing large parts of the lymph node, with extensive extracapsular extension into the adjacent adipose tissue. Immunohistochemical documentation of CD68 expression (clone PGM1, (g)) and absence of ALK1 expression (h). The space bars represent 0.5 cm in (a), 1 mm in (e), and 100 *μ*m in (b)–(d) and (f)–(h).

We describe the case of a 57-year-old lady who presented with hemoptysis.While being initially treated for several weeks for presumed oesophagitis, hemoptysis continued, now associated with slight dyspnea, and the patient complained of a new onset of headaches that radiated into the neck. Bronchoscopy revealed a 1.3 cm endotrachel tumor, located 6 cm above the bifurcation. Histological examination of the superficial endoscopic biopsy suggested an inflammatory myofibroblastic tumor (IMT), but the diagnosis was limited by the paucity of the material. Endoscopic laser resection was incomplete, but relieved the symptoms for a while. A CT scan furthermore provided evidence for bilateral lymphadenopathy and hence sarcoidosis was considered as a putative differential diagnosis. About one month after laser resection, the patient was referred to the thoracic surgery center of the Lausanne University, medical center, where the lesion was entirely resected transthoracically with a 2.1 × 2.2 cm tracheal fragment (Figure 1(a)). Due to its localization in the immediate vicinity of the carina and the intense fibrous reaction to preceding laser treatment, a pedicled latissimus dorsi flap, reinforced with a segment of the 4th rib, was used for reconstruction [1]. In addition, 15 hilar lymph nodes were exercised, some of which measuring up to 1.7 cm in largest dimension. The lady fares well 24 months after the surgery, with no evidence of tumor recurrence or systemic dissemination.

*Histological Examination of the Tracheal Lesion* showed a dense proliferation of spindle cells, partly with an epithelioid aspect, arranged in well-organized fascicles (Figure 1(b)). We appreciated a very mild degree of nuclar atypia, with slightly irregular nuclear contours and small inconspicuous nucleoli. The proliferative activity was low (less than 2 mitoses per 10HPF), confirmed by MIB-1 immunohistochemistry. No atypical mitotic figure was seen. Admixed to the scant stroma were many inflammatory cells, mostly lymphocytes, plasma cells, and rare eosinophils and mast cells. The lesion had a total thickness of 0.5 cm, traversing the tracheal mucosa, but without invasion into the adjacent cartilage (Figure 1(a)). By immunohistochemistry, the tumor cells showed a cytoplasmic decoration with antibodies to CD68 (both KP-1 and PGM1, Figure 1(c)), S-100 and of ALK1 (Figure 1(d)). The cells were negative for smooth muscle actin and desmin, CD1a, CD117, CD21, CD23, CD45, and pan-cytokeratins (not shown). The presence of multinucleated giant cells in the vicinity of the luminal surface of the lesion was ascribed to the preceding interventions (biopsy, laser resection) and the presence of fungi or bacterial colonies was excluded by H&E histology and by special stains (PAS, Grokott, Gram stains, not shown).

*Histological examination of the hilar lymph nodes* revealed a marked granulomatous reaction in every one of 15 sampled nodes. The granulomas were very well delimited, sometimes confluent into large masses, with an admixture of multinucleated giant cells, but with no evidence of caseous necrosis (Figures 1(e) and 1(f)). In most areas, the epithelioid histiocytes exhibited a histomorphological aspect reminiscent of the spindle cells forming the lesion in the trachea. Microorganisms were ruled out by standard H&E histology and using special stains (PAS, Grokott, Gram, not shown). In at least one large lymph node, the capsule was transgressed by the granulomatous reaction, extending widely into the adjacent adipose tissue, focally associated with coagulation necrosis (Figures 1(e) and 1(f)). By immunohistochemistry, the epithelioid cells reacted positively with CD68 (PGM1, Figure 1(g)) and focally with S-100, with an admixture of mostly CD4 positive (T-), less frequent CD20-positive (B-) lymphocytes. No reaction was detected with CD1a, nor with ALK-1 (Figure 1(h)), despite repetition of the reaction in parallel with the tracheal lesion, in which ALK-1 positivity was confirmed (Figure 1(a)).

The dilemma was not so much how to name the two lesions: the spindle cell proliferation in the trachea was diagnosed as inflammatory myofibroblastic tumor (IMT, confirmed by ALK1 positivity and by outside expert consultation by Dr. S. Coffin, Vanderbilt University) and the granulomatous spindly lesions in the adjacent hilar nodes, which differed from the first lesion by their granulomatous appearance and the absence of immunoreactivity for ALK1, were diagnosed as sarcoid-like lesions. The dilemma was rather how to interpret the biology of these two lesions. 

IMTs are rarely metastasizing myofibroblastic neoplasms of low to intermediate malignant potential and predilection for the lung and abdominopelvic region in children or young adults [2, 3]. They tend to recur, in approximately 25% of the cases—in particular in delicate sites such as the trachea, where surgical resection is difficult and hence often incomplete—and show persistent local and invasive growth [2, 4]. The prognosis of IMTs is excellent after complete excision and some tumors may even undergo spontaneous regression [2, 3].

Histologically, the diagnosis of IMT is based on: (i) diffuse inflammatory infiltration of a spindle cell proliferation with predominantly plasma cells (hence the former term “plasma cell granuloma”), (ii) mild nuclear atypia including scattered ganglion-like cells, (iii) low mitotic rate without atypical mitotic figures, and (iv) ALK positivity by immunohistochemistry and ALK gene rearrangement. 

Our case is somewhat special in that a “classic” IMT was accompanied by a massive granulomatous reaction in the hilar lymph nodes. What could be the pathophysiological link between these two co-existing lesions? In the following, we offer five possible explanations, listed in the order from least likely to most likely in the authors' eyes.

1. The lymph node lesions could represent metastases of the tracheal IMT into the hilar nodes. Arguments against this idea are the reported rarity of IMT metastases into single, let alone multiple lymph nodes [5] and the different histomophological appearance and immunostaining pattern (see Figure 1).
2. The tracheal lesions might not be an IMT, but rather extension of the sarcoidosis to the trachea, with aberrant ALK-1 positivity. However, to our knowledge, no ALK-1 positivity has ever been reported in cases of well-documented sarcoidosis.
3. Co-existence of two entirely independent lesions, an IMT in the trachea and a sarcoidosis of the hilar nodes. However, the partly overlapping histomorphology of the two lesions and the minuscule statistical probability of two rather rare lesions coexisting in the same patient, in the same localization, and at the same time, might be advanced as arguments against this option.
4. An as yet unestablished coexistence between a tracheal IMT and a true sarcidosis, in analogy to the well-established coincidence of sarcoidosis and testicular tumors in young men [6]. In support of this idea, we might refer to a case report in the french-speaking medical literature of a coincidence of an inflammatory pseudotumor of the orbit in a 13 year old child with a “suspicion of sarcoidosis” [5].
5. The tracheal IMT inducing a granulomatous, sarcoid-like reaction in the lymph nodes, just as occasionally seen in the dependent nodes of malignant epithelial and hematological tumors [7] as well as in situations of chronic inflammation [8], including asthma [9], aspergilloma [10], and breast implants [11]. These reactive lesions are thought to arise secondary to the proliferative action of a distinct cytokine and growth factor profile [12] and the selective activation of a distinct subset of T-lymphocytes [13]. Even though IMTs are often accompanied by a febrile state and elevated inflammatory markers that may help to imagine such a pathophysiological link, our patient did not present with any signs of inflammation, but rather with symptoms related to mechanical tracheal obstruction and hemorrhage. Finally, we need to consider the theoretical possibility that the initial laser resection of the tumor may have induced a florid granulomatous reaction in the dependent lymph nodes, even though we found no evidence of birefringent or other material (i.e., carbon) in the affected lymph nodes.

Among these five possible explanations, the authors assume that the last version is likely closest to the truth, even though this conclusion remains largely speculative and open to discussions. In this context, the authors would be interested to learn whether any of the distinguished readers of this case report may have made a similar observation and kindly ask him/her to share this information with us in order to better understand this interesting pathophysiological phenomenon.
